# Comparison of Success Rates of Different Methods of Ultrasound-Guided Radial Artery Cannulation (Short-Axis and Long-Axis Methods) Against the Traditional Palpatory Method: A Prospective Randomized Study

**DOI:** 10.5152/TJAR.2021.1364

**Published:** 2022-02-01

**Authors:** Mounica Rajasekar, Senthilkumar Sukumar, Venkatesh Selvaraj

**Affiliations:** Department of Anaesthesiology and Pain Medicine, Sri Ramachandra Institute of Higher Education and Research, Chennai, India

**Keywords:** Blood pressure, cannulation, crossover, radial artery, ultrasound

## Abstract

**Objective:**

Recent meta-analysis comparing the success rates of various methods of arterial cannulation in adult patients found heterogeneity in the available data. Hence, we did this study to evaluate and compare the success rate of palpatory method with that of ultrasound guided methods of radial artery cannulation. The aim is to compare the first-attempt success rate of the palpatory method with that of ultrasound-guided radial artery cannulation techniques, namely, the short-axis out-of-plane and the long-axis in-plane methods.

**Methods:**

This is a prospective, randomized, parallel-arm study. Ninety patients aged from 18 to 50 years (presenting for various surgeries requiring radial artery cannulation for invasive blood pressure monitoring or frequent arterial blood gas analysis) were divided into 3 groups. Each group had one of the 3 techniques of radial arterial cannulation, namely palpatory, short-axis ultrasound method, and long-axis ultrasound method. The parameters analyzed were first-attempt success rate, number of attempts needed, cannulation time, need for crossover of technique, and incidence of complications. Multivariate analysis was done with one-way ANOVA, with Tukey's post hoc test. For categorical data, the chi-square test was used. The probability value of .05 was considered as a significant level.

**Results:**

The first-attempt success rate was 76.7% in the long-axis method, 86.7% in the short-axis method, and 56.7% in the palpatory method. The short-axis method has been shown to have a shorter cannulation time, fewer attempts needed for successful cannulation, and lower need for crossover of techniques when the first 2 attempts fail.

**Conclusions:**

: We conclude that the ultrasound-guided short-axis method of radial artery cannulation is associated with higher first-attempt success rate compared to the traditional palpatory method.

**Main Points**

- The Short-axis out-of-plane ultrasound-guided radial artery cannulation method has a higher first-attempt success rate than the palpatory method.

- It has also a shorter cannulation time and fewer attempts are needed for successful cannulation.
- Bedside ultrasound is an effective tool for successful cannulation of the radial artery.

## Introduction

Intra-arterial cannulation for continuous beat-to-beat blood pressure monitoring and frequent arterial blood gas analysis has become an essential component for physiological optimization and anaesthetic management of patients with significant perioperative morbidity and/or in patients undergoing surgery with major blood loss. The radial artery is the most preferred site for arterial cannulation.^1^ This invasive procedure is generally safe, but in approximately 0.09%^2^ of patients, it is associated with permanent ischemic complications. Recent literature reviews have shown that ultrasound guidance provides the ability to overcome the factors that cause cannulation failure through real-time visualization of the artery, thus increasing the success rates of cannulation.^3^ Two different techniques are available for vascular visualization, namely the long-axis in-plane (LA-IP) approach and the short-axis out-of-plane (SA-OOP) approach.^4^ Recent literature review^5^ has shown that there is a lot of heterogeneity in the data in studies that have compared the traditional palpatory method with the 2 ultrasound-guided approaches of radial artery cannulation in terms of the first-attempt success rate of cannulation.

We hypothesized that the SA-OOP technique of ultrasound-guided radial artery cannulation would be more successful at the first attempt than the other 2 methods of radial artery cannulation. Hence, this study was conducted to compare the success rates of the different methods of ultrasound-guided radial artery cannulation (the short-axis and long-axis methods) against the traditional palpatory method. Furthermore, the time taken for cannulation, the number of attempts, and the complication rates were also assessed between the groups as secondary outcomes of the study. 

## Methods

After Institutional Ethical Committee approval, clinical trial registry (CTRI/2019/02/017749), and informed patient consent, this prospective, randomized study was conducted on 90 adult patients aged from 18 to 50 years presenting for various surgeries requiring radial artery cannulation for invasive blood pressure monitoring or frequent arterial blood gas analysis in the course of the preoperative management. The need for radial artery cannulation was assessed during routine preoperative assessment and the procedure was explained to the patients. The exclusion criteria were the following: any signs of infection near the puncture site, recent arterial cannulation at the same site during this hospital admission, hemodynamically unstable patients, history or evidence of peripheral vascular disease, and coagulopathies. Patient dropouts were expected due to patient refusal after recruiting and a negative modified Allen’s test (if the hand does not flush within 15 seconds after release of ulnar occlusive pressure)taken as inadequate collateral flow.

The 90 patients enrolled were block-randomized into 1 of the 3 groups (30 in each group) using computer-generated randomization numbers concealed using the sealed envelope technique. On the patient’s arrival in the operating room, monitors like ECG, NIBP, and pulse oximeter were connected and the baseline values were recorded. The left median cubital vein was used to establish venous access in all the patients. The hockey stick probe of the bedside USG machine (Sonosite R ultrasound system, Sonosite Inc., Bothell, WA, USA) was used with strict aseptic precautions. All the radial artery cannulations were done by the same experienced anaesthesiologist who had prior experience in ultrasound-guided vascular cannulation. All the arterial cannulations were done prior to the induction of anaesthesia, that is, in a conscious patient, with local anaesthetic skin infiltration at the puncture site. In all patients, the non-dominant wrist was extended at an angle of 30° on a splint to keep the angle of the wrist unchanged. In all patients, hand hygiene was ensured before gloving and a sterile barrier was placed. Skin preparation was done with alcohol-based chlorhexidine antiseptic. A 20G intravenous access cannula (Jelco^R^, Smiths Medical International Ltd, Kent, UK) was used to cannulate the artery in all the patients.

## Radial Artery Cannulation Technique

### Group A Long-Axis In-Plane Ultrasound Technique

A transducer was placed along the axis of the radial artery in the radial aspect of the distal forearm. The radial artery was identified by the lack of compressibility and visible pulsations. The skin was infiltrated with 1 mL of 2% lignocaine. After identifying the radial artery in the long axis, a 20G cannula was used to cannulate the radial artery. Entry into the artery was confirmed by visualizing the backflow of blood into the hub of the cannula. The cannula was then slowly advanced over the stylet by rotating movements, and was connected to the pressure transducer which had been kept ready; placement was confirmed after visualizing the arterial waveform on the monitor.

### Group B Short-Axis Out-of-Plane Ultrasound Technique

With the initial preparation as the long-axis technique, in these patients, the transducer was placed in the radial aspect of the distal forearm in an axis transverse to the radial artery. The skin was infiltrated with 1 mL of 2% lignocaine. After identifying the radial artery in the short axis, a 20G cannula was used to cannulate the radial artery. The cannula was then slowly advanced over the stylet by rotating movements. 

### Group C Traditional Palpatory Method

In this method, the radial artery was cannulated by the traditional palpatory method. To eliminate the influence of other factors in the palpatory method on the success rate and the procedure time, the use of guidewire or transfixation was not allowed in the study population. Once the cannulation was done, the extension was connected immediately to obtain the arterial waveform on the monitor.

### The Parameters Observed

The first-attempt success rate of radial artery cannulation between the 3 groups was observed as the primary outcome measure. The secondary outcome measures were: number of attempts needed for successful cannulation, time taken for cannulation, need for crossover between techniques, and complications if any.

The definitions of the various outcome measures are:

Successful cannulation**:** defined as an attempt at arterial cannulation by any of the 3 methods and achieving an endpoint of arterial waveform on the monitor.

One attempt at cannulation: defined as skin puncture leading to successful cannulation or removal of the cannula from the skin after an unsuccessful attempt (redirection is taken as the same single attempt).

Time taken for cannulation: Time taken from the start of skin puncture to the appearance of arterial waveform on the monitor after transducing the cannula.

Need for crossover: After 2 unsuccessful attempts, crossover between the 3 techniques or personnel is allowed and noted.

The following are procedure-related complications: 

Hematoma: Appearance of a visible swelling at the site of cannulation during the attempt. Vasospasm: Inability to feel the pulse clinically by the anaesthesiologist following unsuccessful attempts at cannulation and distal to the site of cannulation. 

## Statistical Analysis

All data were collected by the same anaesthesiologist from all the patients. All the patients were successfully cannulated according to the study protocol. There were no dropouts from the study. The statistician analyzing the data was unaware about the type of cannulation technique utilized in the 3 groups. The collected data were analyzed with the Statistical Package for Social Sciences (SPSS) version 23.0 software (IBM Corp.; Armonk, NY, USA). To describe the data with descriptive statistics, frequency analysis and percentage analysis were used for categorical variables and the mean and standard deviation were used for continuous variables. Distribution was tested using the Shapiro–Wilk normality test. To find the significant difference in the multivariate analysis, one-way ANOVA with Tukey's post hoc test was used. To find the significance in categorical data, the chi-square test was used. In all the above statistical tools, the probability value of .05 was considered as a significant level.

We calculated the sample size based on a pilot case with 10 cases in each group, with a first-attempt success rate of 51% and 86% between the 2 methods, an effect size of 35%, a power of 80%, type-I error 0.05, a sample size of 28, and an expected dropout rate of 10%. The sample size for each arm was calculated as 30 in each group.

**Figure 1.**
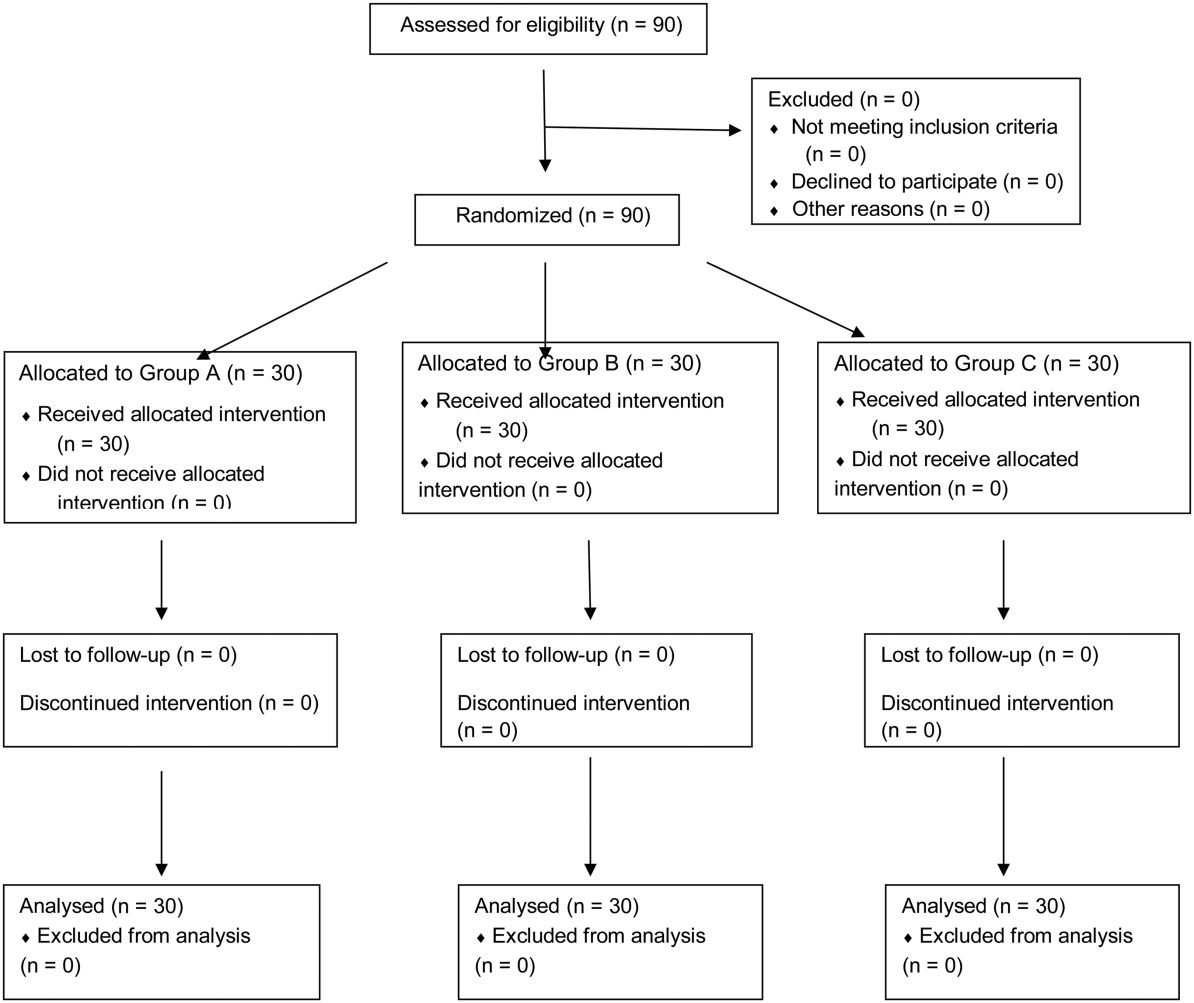
CONSORT flowchart.

All the patients recruited completed the study protocol and there were no dropouts at the end of the study, as shown in the Figure 1.

## Results

Analysis of the study population shows that there was no statistical difference in terms of mean age, sex distribution, and body mass index (BMI) among the 3 groups, as shown in Table 1. The differences in distribution of the study population between the various age groups are also statistically insignificant. The baseline parameters like heart rate and the systolic and diastolic blood pressure are comparable between the 3 groups, and there is no statistically significant difference among the 3 groups, as seen in the Table 2. The first-attempt success rate of radial artery cannulation was higher with the ultrasound-guided short-axis method when compared to that of the palpatory method, and the values were statistically significant, as shown in Table 3. Also, the first-attempt success rate of radial artery cannulation was higher with the ultrasound-guided long-axis method when compared to the palpatory method, and the values were statistically significant.

Though the first-attempt success rate was more with the ultrasound-guided short-axis method (86.7%) when compared to the long-axis method (76.7%), the values were not statistically significant. The mean values of cannulation time in the palpatory, long-axis, and short-axis approach were 52.33, 45.07, and 35.07 seconds respectively, which is statistically significant among the 3 groups, as shown in Table 4. Hence, the short-axis method takes less time for successful cannulation at first attempt, compared to the other 2 methods. When comparing the need for a third attempt or the need for crossover between the techniques, the short-axis method demonstrates less need for crossover when compared to the other 2 methods, as shown in Table 4. There is no statistically significant difference with respect to complications (hematoma formation, vasospasm) among the 3 groups, as shown in Table 4. 

## Discussion

The primary finding of this study is that the short-axis USG method has a significantly greater first-attempt success rate than the traditional palpatory method. The short-axis method was also found to have a higher first-attempt success rate than the long-axis method, though statistically insignificant. This ultrasound guidance is particularly so helpful because it has shown benefit in subjects with variable diagnoses approaching the emergency department for treatment in the hands of trainee physicians.^6^ Ultrasound guidance also has been shown to reduce the number of attempts and increase the first-attempt success rate for radial artery cannulation.^7,8^ The ultrasound has been shown to be handy not only in adult patients, but also in varied groups of patients, for successful radial arterial cannulation with different levels of performer expertise.^6,9,10^ There are 2 basic needling approaches using ultrasound (the short-axis out-of-plane approach and the long-axis in-plane approach) both of which have their own merits and demerits.^11^ Our study was unique in the way that all the 3 methods, namely palpatory, short-axis, and long-axis ultrasound methods of radial arterial cannulation were compared together in the same study. The meta-analysis also consistently showed heterogeneity in the data collected and none of the analyses could infer a consistent conclusion, except that USG helps in achieving a higher first-attempt success rate. Most of the earlier studies have been attempted in patients under general anaesthesia, which the authors realized could be confounding the outcome because of the effect of the general anaesthetic on the radial artery diameter.^12^ Hence, the patients in our study were kept conscious, and the study was done using local anaesthetic infiltration.

A recent meta-analysis^5^ involving 10 studies of ultrasound-guided radial artery cannulation has shown improved overall success rate of radial artery cannulation, and a decrease in time to successful cannulation or in the mean number of cannulation attempts, over the digital palpation technique. Similar to our study, they have inferred that there is no significant clear-cut advantage of the short-axis method over the long-axis method in terms of successful cannulation. 

As a unique feature, our study addressed the factor of aging, which is associated with alterations in a number of structural and functional properties of arteries, including the diameter, wall thickness, wall stiffness, and endothelial function.^13, 14^ Hence, we grouped the participants according to age-group intervals and analyzed the distribution of patients in various age intervals between the groups, which was found to be statistically insignificant. The earlier studies have included patients of varied age groups–a confounding factor because of the age-related changes in the artery structure. 

The second uniqueness in our study is that we defined cannulation failure as the need for more than 3 attempts to achieve successful cannulation. Also, our study gave scope for the anaesthesiologist to crossover to the alternate method in case of failure of the initial 2 attempts. The authors felt that it would be unethical to try more than twice at the same site, and hence, provided scope for changing the technique at the third attempt at the discretion of the anaesthesiologist. The authors also felt that because of this design, the study has achieved successful attempts in all the participants.

The third uniqueness about our study is that we have taken the appearance of arterial waveform on the monitor as the end point for successful cannulation, unlike in the earlier studies. The authors felt that simple aspiration of a gush of blood would not be sufficient to confirm a successful arterial cannulation and invasive monitoring. Earlier studies have not stated the precise endpoint of successful arterial cannulation.

Hence, in this study, we found that the short-axis ultrasound method has a higher first-attempt success rate, required fewer attempts, and shorter cannulation time than the traditional palpatory method. When compared to the long-axis USG method, the short-axis ultrasound method approach has a higher first-attempt success rate clinically, fewer number of attempts, and shorter cannulation time. 

Our study has some limitations. First, blinding was not possible in our study. Secondly, the ultrasound evaluation of the distal flow would have added much more information to the secondary outcomes. Morbidly obese patients and those with acute hemodynamic changes were excluded from the study. If those patients had been included, the effectiveness of ultrasound could have been more widely evaluated.

Technically, the authors felt that the arterial cannulation procedure requires 2 steps: first, locating the artery with the stilletted cannula; and once it enters the artery and is sufficiently inside, moving the cannula sheath over the stationed stillette. The ultrasound short-axis view should be of greater help in locating the artery, which is the initial part of the cannulation procedure, and the long-axis view is more useful in visualizing the path of the cannula moving inside the artery.

Our study has shown the scope for further research in this area of interest. Future studies have to specifically evaluate the use of ultrasound guidance in difficult radial artery catheterization, as in patients with severe hypotension or morbid obesity, in addition to studying the integrated use of both methods of ultrasound for effective arterial cannulation.

## Conclusion

We conclude that ultrasound-guided short-axis method of radial artery cannulation is associated with a higher first-attempt success rate compared to the traditional palpatory method. The ultrasound-guided short-axis method has a higher first-attempt success rate than the long-axis method, though statistically insignificant. The short-axis method tends to have a shorter cannulation time and fewer attempts are needed for successful cannulation.

## Figures and Tables

**Table 1.** Comparison of Demographic Profile Between Groups

	Groups			
Parameters	Palpatory	USG Long Axis	US Short Axis	*P*
Age (years) <40 n (%)	7 (23.3)	6 (20.0)	6 (20.0)	.778
Age (years) 41-50 n (%)	16 (53.3)	15 (50.0)	19 (63.3)	
Age (years) 51-60n(%)	7 (23.3)	9 (30.0)	5 (16.7)
Male n (%)	21 (70)	20 (66.7)	20 (66.7)	.950
Female n (%)	9 (30.0)	10 (33.3)	10 (33.3)
Body Mass Index	26.70	26.97	26.70	.912

No statistical significance at *P*-value > .05.

USG, ultrasound.

**Table 2.** Comparison of Hemodynamic Parameters Between the Three Groups

	Palpatory	USG Long Axis	US Short Axis	*P*
Heart rate (beats/min)	72.13	71.87	72.67	.639
Systolic BP (mm Hg)	150.77	147.47	146.90	.597
Diastolic BP (mm Hg)	90.70	89.07	90.43	.421

No statistical significance at *P*-value > .05.

USG ultrasound.

**Table 3.** Comparison of First-Attempt Success Rate Between Groups

	Groups					
No. of Attempts	Long-Axis Method n (%)	Short-Axis Method n (%)	Palpatory Method n (%)	Long Axis Versus Short Axis	Long Axis Versus Palpatory	Short Axis Versus Palpatory
1	23 (76.7)	26 (86.7)	17 (56.7)	0.052	0.037	0.01
2	5 (16.7)	3 (10.0)	8 (26.7)			
3	2 (6.7)	1(3.3)	5 (16.7)

Statistical significance at *P-*value < .05

**Table 4.** Comparison of Other Outcome Parameters Between the Three Groups

	Groups				
Parameters	Palpatory Mean+/-SD	Long-Axis Method	Short-Axis Method	*F*	*F*
Time taken for cannulation (in seconds)	52.33 ± 7.15	45.07 ± 8.29	37.53 ± 10.77	20.904	.01
Need for crossover (n, %)	5 (16.7%)	1 (3.3%)	2 (8.9%)	3.567	.17
Hematoma (n, %)	3 (10.0%)	1 (3.3%)	1(3.3%)	4.068	.67
Vasospasm (n)	3	3	6	4.028	.59

Statistical significance at *P-*value < .05
